# Landscape Patterns Drive Functional Diversity of Macroinvertebrate Communities Along the Elevation Gradient in the Chishui River

**DOI:** 10.3390/biology14091149

**Published:** 2025-08-31

**Authors:** Xiaopeng Tang, Zhenhao Liu, Fei Liu, Yun Cheng, Tingsong Yu, Xuehua Li, Qiang Qin, Fubin Zhang

**Affiliations:** 1 College of Environmental Science and Engineering, China West Normal University, Nanchong 637009, China; tangxiaopeng616219@163.com (X.T.); lzh18783900795@163.com (Z.L.); qiangqin710@163.com (Q.Q.); 2 Institute of Hydrobiology, Chinese Academy of Sciences, Wuhan 430072, China; liufei@ihb.ac.cn; 3 Zhaoyang District Water Bureau, Zhaotong 657000, China; ztazj@126.com; 4 Yunnan Management and Conservation Bureau of National Nature Reserve of Rare and Endemic Fishes in the Upper Yangtze River, Zhaotong 657000, China; songeryu188@163.com (T.Y.); ynghjlxh@163.com (X.L.)

**Keywords:** biodiversity conservation, landscape heterogeneity, landscape fragmentation, functional traits, freshwater ecology, the Chishui River

## Abstract

Landscape patterns serve as important drivers of macroinvertebrate biodiversity. However, the mechanisms through which landscape dynamics influence biodiversity across different elevation gradients in undammed rivers remain poorly understood. Here, this study investigated macroinvertebrate communities in the Chishui River, which represents the only undammed tributary maintaining a natural flow regime in the upper Yangtze River. We documented 97 macroinvertebrate taxa (3 phyla, 16 orders, and 57 families) with a mean density of 314.93 ind./m^2^. NMDS and PERMANOVA analyses revealed significant spatial heterogeneity in macroinvertebrate community composition, but no overall seasonal variation. However, functional diversity indices (e.g., FRic) exhibited seasonal fluctuations across the river system. Furthermore, we assessed nine landscape metrics to capture heterogeneity, complexity, and fragmentation effects. Random forest modeling with nine predictors revealed that landscape heterogeneity primarily drove functional diversity in the upstream areas, whereas landscape fragmentation was the dominant factor in the downstream areas. Functional diversity, which reflects trait-based ecological roles, provides more direct insights into ecosystem processes than taxonomic diversity alone. Notably, the taxonomic diversity indices (Margalef richness, Shannon–Wiener diversity, Simpson diversity, and Pielou evenness index) showed no significant correlations with landscape metrics. These findings highlight the critical role of functional diversity in evaluating landscape-mediated ecological effects. For effective conservation, management strategies should prioritize reducing anthropogenic disturbances in downstream areas while preserving natural landscape heterogeneity.

## Simple Summary

Understanding how landscape patterns affect biodiversity is crucial for river ecosystem conservation. In this study, we investigated the relationship between landscape characteristics and macroinvertebrate communities in an undammed river. Our results showed that macroinvertebrate communities varied significantly across different river sections but remained stable across seasons. Notably, landscape heterogeneity was the key driver of functional diversity in upstream areas, whereas downstream communities were more affected by landscape fragmentation. These findings highlight the need for region-specific conservation strategies to maintain river biodiversity.

## 1. Introduction

Biodiversity maintains critical ecosystem functions, including ecosystem productivity, stability, and nutrient cycling, which can collectively mitigate global environmental changes [1,2]. However, anthropogenic land-use changes have driven extensive ecosystem degradation and accelerated biodiversity decline [3,4], and profoundly reduced habitat heterogeneity in the landscape [5]. This may induce indirect cascading effects within biotic communities through trophic downgrading and functional group simplification [6]. Moreover, environmental filters strongly regulate species distributions and community assembly [7]. Landscape heterogeneity represents a fundamental driver of habitat diversity [8]. Increased heterogeneity expands ecological niche spaces, thereby potentially enhancing species richness [9].

Landscape patterns, including patch size, shape, composition, and configuration arising from heterogeneous land-use types, which collectively function as the primary drivers of biodiversity [10,11]. Therefore, landscape configuration constitutes a robust indicator for evaluating biodiversity responses to environmental change. However, elucidating the mechanistic links between landscape pattern dynamics and biodiversity responses remains a significant challenge. Identifying the most reliable assessment methods also poses a critical question for ecologists and managers [12].

Traditional biodiversity assessments have predominantly relied on taxonomic metrics, particularly species richness, a foundational and interpretable measure of ecosystem complexity [13,14]. However, these conventional approaches suffer from critical limitations. Specifically, they assume functional equivalence among species yet fail to account for interspecific trait variability [15]. Recent advances in community ecology emphasize that functional trait diversity provides more mechanistic insights into biodiversity responses to environmental change [16].

Functional traits are defined as species-specific physiological, morphological, and behavioral attributes affecting fitness [17]. These traits mediate both species’ environmental adaptations and their ecosystem roles [18]. Unlike taxonomic approaches, trait-based analysis provides mechanistic insights into aquatic ecosystem dynamics by directly linking organismal characteristics to ecosystem functioning [19].

Functional trait-based methods offer a robust framework for investigating species responses to environmental stressors [20]. By quantifying community-level functional variation, these methods can be used to elucidate ecological processes that remain obscured in taxonomic analyses [17]. This approach advances both theoretical understanding of biodiversity–ecosystem functioning relationships and provides actionable insights for evidence-based conservation.

Functional diversity quantifies the spatial distribution of community functional traits [11], providing three key ecological insights. First, it reveals the underlying ecological mechanisms more effectively than taxonomic approaches by incorporating functional trait data [21]. Second, functional diversity bridges taxonomic diversity and ecosystem processes through resource utilization pathways [22]. Finally, it serves as an ideal metric for landscape pattern analyses [23,24]. The taxonomic–functional diversity relationship is context-dependent and strongly mediated by landscape patterns [25]. Consequently, integrating these two dimensions of diversity is essential for elucidating biome responses to landscape change [18].

Here, we selected the Chishui River (CSR) as the study area. The CSR is the only large-scale tributary in the upper Yangtze River that remains undammed. It retains pristine hydrological conditions and serves as a critical refuge for aquatic biodiversity conservation [26]. Characterized by a complex topography and high landscape heterogeneity [27], the basin offers an ideal system to examine macroinvertebrate diversity responses to landscape patterns.

Macroinvertebrates serve as particularly valuable ecological indicators in freshwater ecosystems, offering two key advantages for environmental assessment [28,29]. First, macroinvertebrates mediate essential ecosystem processes, such as material cycling and energy flux [30]. Their rapid responses to habitat alteration further establishes them as reliable bioindicators [24,31]. Second, their sensitivity to both local environmental conditions and landscape-scale disturbances enables comprehensive ecosystem evaluation [32]. The CSR exhibits a unique undammed hydrology that is prone to seasonal flooding [33]. Additionally, it features a stark landscape gradient, ranging from karst-dominated upper reaches to intensively farmed and urbanized lower basins with degraded riparian vegetation [34]. These dynamics establish macroinvertebrates as valuable ecological bioindicators.

Recent advances have highlighted functional diversity approaches as particularly powerful for understanding landscape–macroinvertebrate relationships [35,36,37]. As anthropogenic landscape modification intensifies globally, elucidating these mechanistic linkages through functional traits has become critical for understanding ecological systems.

Previous studies of the CSR have focused on the macroinvertebrate species composition [38,39]. Nevertheless, the mechanisms driving functional diversity in this system remain poorly understood. Furthermore, existing studies on landscape pattern effects have been limited to single elevation gradients (e.g., high-elevation regions [28] or low-elevation regions [40]). This narrow focus may limit the generalizability of findings across different altitudinal zones and hinder a comprehensive understanding of elevation-dependent ecological processes. Systematic comparisons of landscape pattern effects across elevation gradients remain scarce.

The CHR provides an ideal natural laboratory for addressing this gap. It exhibits a remarkable elevational gradient, ranging from 2237 m at its headwaters to 205 m at the river mouth [33]. This study aims to (1) characterize the spatiotemporal distribution patterns of macroinvertebrate communities in the CSR basin; (2) evaluate whether taxonomic or functional diversity better reflects landscape-driven ecological effects; and (3) identify key landscape drivers of functional diversity in the upstream (high elevation) and downstream (low elevation) areas. This study will complement existing research on macroinvertebrate functional diversity in the CSR basin. Additionally, it provides new insights into the mechanisms through which landscape patterns influence biodiversity across different elevation gradients.

## 2. Materials and Methods

### 2.1. Study Area and Sampling Sites

The CSR (27°20′–28°50′N, 104°45′–106°51′E) originates in Zhenxiong County, Yunnan Province, flowing 436.5 km through Yunnan, Guizhou, and Sichuan provinces before joining the Yangtze River at Hejiang County. Encompassing a watershed area of 20,440 km^2^, it remains the last undammed primary tributary in the upper Yangtze basin [26]. The basin experiences a subtropical monsoon climate with marked seasonal hydrologic variability [39]. Moreover, the mean annual temperatures range from 11 to 13 °C, with 800–1200 mm of precipitation falling predominantly (60%) during the June–September monsoon season [38,41].

This watershed features complex topography dominated by karst landforms, including mountains, hills, and deep valleys [33]. The primary land uses are forestland and cropland, creating a distinct natural landscape [42]. This high environmental heterogeneity of the basin makes it an ideal system for investigating how landscape patterns influence macroinvertebrate biodiversity across multiple dimensions.

**Figure 1:**
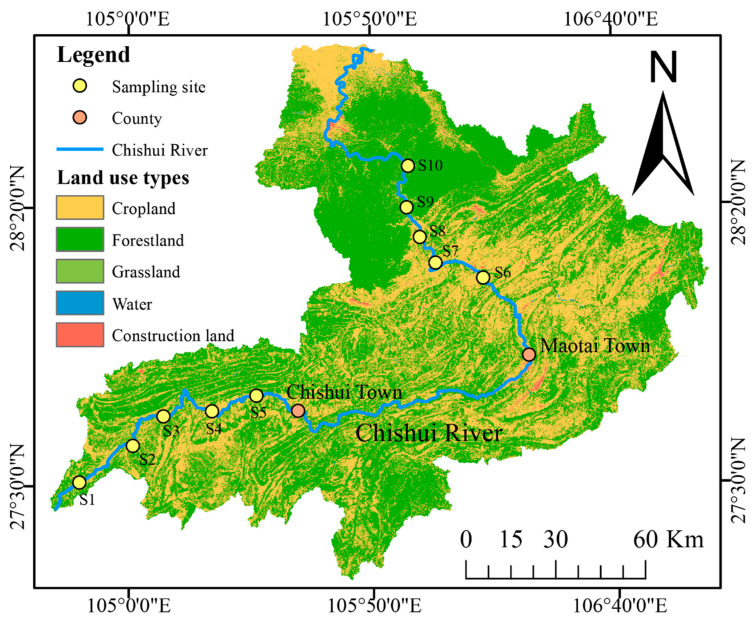
Land-use composition and spatial configuration of sampling sites along the CSR.

For this study, the CSR was stratified into upstream, midstream, and downstream areas based on elevation gradients, topography, vegetation, and hydrography, with the boundaries delineated by the towns of Chishui and Maotai [26]. Ten sampling sites were established across the basin (Figure 1): five in the upstream area (S1–S5; mean elevation: 1324 m) and five in the lower reaches (S6–S10; mean elevation: 300 m).

### 2.2. Macroinvertebrate Sampling

Based on hydrological data (2013–2020) from our prior study in the CSR [27], the water level and water flow were lowest in November, highest in August, and intermediate in April. Accordingly, we defined the seasons as follows: spring (baseflow season), summer (wet season), and autumn (dry season).

Macroinvertebrate sampling was conducted in April (baseflow season), August (wet season), and November 2024 (dry season). We selected 100 m river reaches at each sampling point to encompass representative habitat characteristics, with particular attention to substrate composition, water depth gradients, and flow velocity regimes. Three quantitative samples and one qualitative sample were collected per site. We used a Surber sampler (420 μm mesh, 0.09 m^2^ area) for wadeable rocky reaches, and a Peterson mudpicker (1/16 m^2^) for non-wadeable silty reaches. To complement and enhance our quantitative assessments, we conducted supplementary qualitative sampling using a D-frame kick net.

All collected samples were processed through 450 μm copper mesh sieves, thoroughly rinsed, and sorted in white sorting trays. We preserved the specimens in 75% ethanol (Guizhou Xinyuan Biotechnology Co., Ltd., Guiyang, China) using 50 mL storage vials. Thereafter, taxonomic identification at the genus or species level was performed using relevant studies [43,44,45]. Ethical approval for the specimen collection was obtained from the Ethics Committee of China West Normal University (Approval No. CWNU2023D002).

### 2.3. Biodiversity Indices

In this study, multidimensional indices were used to assess the biodiversity characteristics of the macroinvertebrate communities. For the taxonomic diversity (TD) assessment, four established indices were computed using the “vegan” package (version 2.6.6.1) in R [46]: the (1) Margalef richness index, (2) Shannon–Wiener diversity index, (3) Simpson diversity index, and (4) Pielou evenness index [47].

For functional diversity (FD), we selected four key indices: functional richness (FRic), functional evenness (FEve), functional divergence (FDiv), and Rao’s quadratic entropy (RaoQ) [48]. Calculations were performed using the “dbFD” function in the “FD” package (version 1.0.12.3). Based on a previous study [41], we selected 10 functional traits (see Table S1 in Supporting Information) representing key ecological strategies, including voltinism, occurrence in drift, swimming ability, morphological adaptations, and habitat preferences.

Furthermore, to identify ecologically significant taxa within the community, we applied a dominance threshold of >0.02 following established ecological criteria [49]. Species exceeding this threshold were classified as dominant. The formula is as follows: *Y* = (*n_i_*/*N*) × *f_i_*, where *n_i_* is the number of individuals of species *i*; *N* is the number of individuals of all species; *f_i_* is the proportion of the number of individuals of species *i* to the total number of individuals.

### 2.4. Landscape Pattern Indices

We derived land-use data for the CSR from the China Land Cover Dataset (CLCD) [50]. The China Land Cover Dataset (CLCD) provides annual 30 m resolution land-use/cover maps from 1990 to 2023. For this study, we used the most recent available data (2023) to ensure temporal relevance. Using ArcGIS 10.7, we reclassified the original data into five categories according to the method of Tang et al. [27]: cropland, forestland, grassland, water, and construction land.

We conducted spatial analyses by delineating sub-watersheds for each sampling site (ArcGIS 10.7) and computing landscape pattern metrics (FRAGSTATS v4.2.1 [51]). These metrics were grouped into three ecological dimensions [11]: (1) fragmentation (number of patches (NP), total edge (TE), and edge density (ED)); (2) complexity (landscape shape index (LSI), largest patch index (LPI), and interspersion juxtaposition index (IJI)); and (3) heterogeneity (patch richness (PR), Shannon’s diversity index (SHDI), and the Simpson’s evenness index (SHEI)). Specific descriptions of these indices are provided in Table S2 (Supporting Information).

### 2.5. Statistical Analysis

Here, we quantified the spatiotemporal variation in macroinvertebrate diversity and tested its relationship with landscape metrics using a suite of statistical approaches. First, the data were tested for parametric assumptions prior to the analysis. The Shapiro–Wilk test was used to assess normality, with *p* ≥ 0.05 indicating a normal distribution. Homogeneity of variance was evaluated using Levene’s test, where *p* ≥ 0.05 confirmed equal variances. Since both assumptions were met (*p* ≥ 0.05 for all key variables, including diversity indices), parametric tests (ANOVA and *t*-tests) were deemed appropriate for the analysis. One-way analysis of variance (ANOVA) was employed to assess differences in macroinvertebrate taxonomic diversity (TD) and functional diversity (FD) across the three hydrological seasons (baseflow season, wet season, and dry season). Subsequently, independent samples *t*-tests were employed to evaluate differences in TD and FD between the upstream and downstream areas.

To quantify the spatiotemporal variation in community structure, non-metric multidimensional scaling (NMDS) was performed on the Bray–Curtis similarity matrix. Model adequacy was evaluated using stress values, with stress < 0.2 indicating reliable ordination. Additionally, a permutational analysis of variance (PERMANOVA) was conducted to quantify the contributions of the hydrological period and spatial location (upstream/downstream) to community structure variation. Statistical significance was determined using 999 permutations.

We employed a two-stage analytical approach to examine landscape–biodiversity relationships. First, Mantel tests assessed spatial correlations between landscape pattern indices and both the TD and FD of macroinvertebrates. Subsequently, we applied random forest (RF) modeling (*n* = 1000 trees) to quantify the relative influence of upstream versus downstream landscape metrics in explaining TD and FD variation. The model was trained using bootstrap-generated datasets, and less important variables were eliminated via backward feature selection. Model accuracy and the significance of retained variables were evaluated using out-of-bag (OOB) samples.

All statistical analyses were conducted with a significance threshold of *α* = 0.05. Parametric tests (one-way ANOVA and *t*-tests) were performed using SPSS 22.0. Multivariate analyses, including NMDS, PERMANOVA, and Mantel tests, were conducted using the “vegan” (version 2.6.6.1) and “linkET” packages (version 0.0.7.4). We performed RF analyses in R (version 4.4.0) using the “randomForest” package [52].

## 3. Results

### 3.1. Composition and Spatial–Temporal Dynamics of Macroinvertebrate Communities

Three systematic surveys conducted in 2024 recorded 97 macroinvertebrate taxa, distributed across 3 phyla, 16 orders, and 57 families. Arthropoda dominated the assemblage (80 taxa, 82.5%), followed by Mollusca (10 taxa, 10.3%) and Annelida (7 taxa, 7.2%).

**Figure 2:**
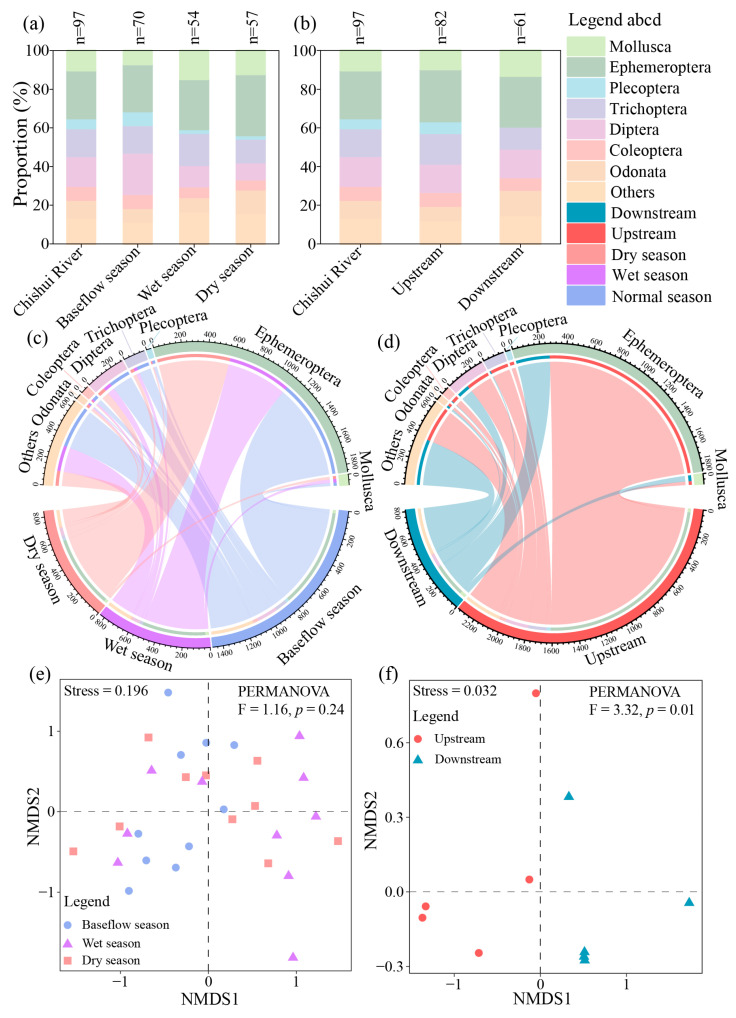
Spatial and temporal variations in macroinvertebrate communities in the CSR. (**a**) Order-level richness across hydrological seasons; (**b**) order-level richness along longitudinal segments; (**c**) seasonal density visualized by order-level chord diagram; (**d**) longitudinal density visualized by order-level chord diagram; (**e**) NMDS ordination by hydrological period; (**f**) NMDS ordination by stream segment.

The macroinvertebrate species richness exhibited seasonal and spatial variations. Peak richness occurred during the baseflow season (70 taxa), exceeding values recorded in the wet season (54 taxa) and dry season (57 taxa) (Figure 2a). Spatially, the upstream areas supported significantly more taxa (82) than the downstream areas (61) (Figure 2b).

Furthermore, the mean macroinvertebrate density across the basin was 314.93 ind./m^2^. Seasonally, the density trend followed the order of baseflow season > dry season > wet season (Figure 2c). Spatially, densities were significantly higher in the upstream areas than in the downstream areas (Figure 2d).

These results indicate that the composition and spatial distribution of macroinvertebrate communities in the CSR are influenced by hydrological seasonality and the longitudinal gradient. The upstream reaches exhibit a greater species richness and abundance, corresponding to both higher habitat heterogeneity and lower anthropogenic pressure. By contrast, seasonal variations in community structure are driven primarily by hydrological regulation.

Additionally, this study revealed some seasonal changes in the dominant taxa (Table S3 in Supporting Information). Among them, the dominance peaked during the baseflow season, led by *Heptagenia* sp., *Ameletus* sp., *Ephemera* sp., *Potamanthus* sp., *Baetis* sp., *Caenis* sp., Chironomidae larvae, and *Saldula* sp. However, the dominant taxa were consistent between wet and dry seasons, including *Heptagenia* sp., *Potamanthus* sp., *Baetis* sp., Chironomidae larvae, and *Neocaridina denticulata*.

The dominant taxa exhibited significant longitudinal variation (Table S4 in Supporting Information). The upstream areas supported eight dominant taxa, compared to seven in the downstream areas. Notably, *Ameletus* sp., *Ephemera* sp., and *Glossophonia* sp. were exclusively found upstream. *Caenis* sp. and *Saldula* sp. only occurred downstream.

The NMDS and PERMANOVA analyses revealed distinct spatial, but not seasonal, patterns in community structure. The hydrological period had no significant effect (F = 1.16, *p* = 0.24; Figure 2e and Table S5 in Supporting Information), whereas the upstream and downstream areas showed statistical differences (F = 3.32, *p* = 0.01; Figure 2f and Table S6 in Supporting Information). These results demonstrate that the macroinvertebrate communities in the CSR are primarily shaped by altitudinal gradient-driven habitat heterogeneity rather than seasonal hydrological variation.

### 3.2. Spatial and Temporal Patterns of Biodiversity

**Figure 3:**
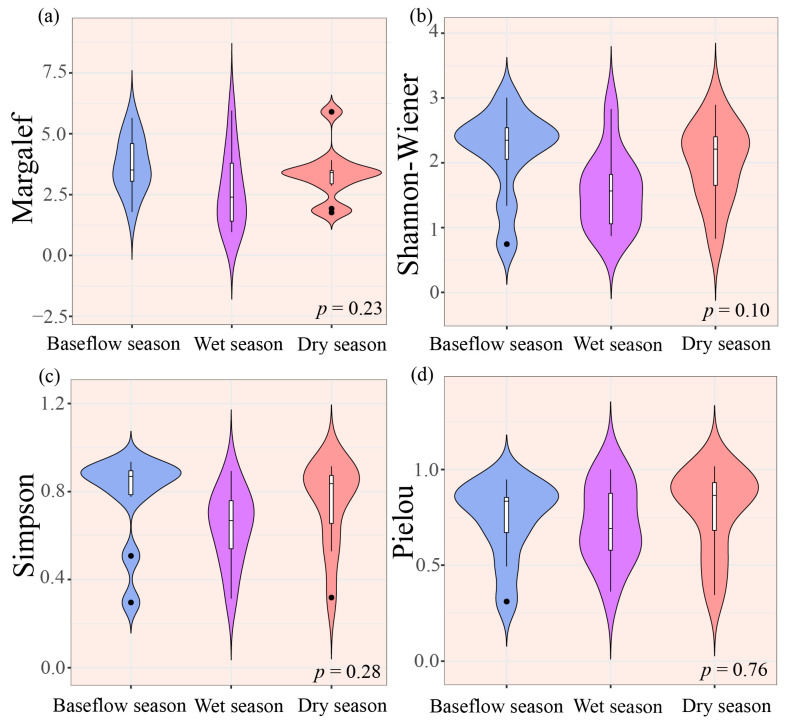
Seasonal variation in macroinvertebrate taxonomic diversity indices. (**a**) Margalef richness index, (**b**) Shannon–Wiener diversity index, (**c**) Simpson diversity index, and (**d**) Pielou evenness index. Note: In the box plot, the central horizontal line represents the median value. The whiskers extend to 1.5× the interquartile range (IQR), and the box boundaries indicate the 25th and 75th percentiles.

One-way ANOVA revealed no significant seasonal variation in taxonomic diversity index (Margalef richness index, Shannon–Wiener diversity index, Simpson diversity index, and Pielou evenness index) (*p* > 0.05, Figure 3). Nevertheless, all indices reached their minima during the wet season.

**Figure 4:**
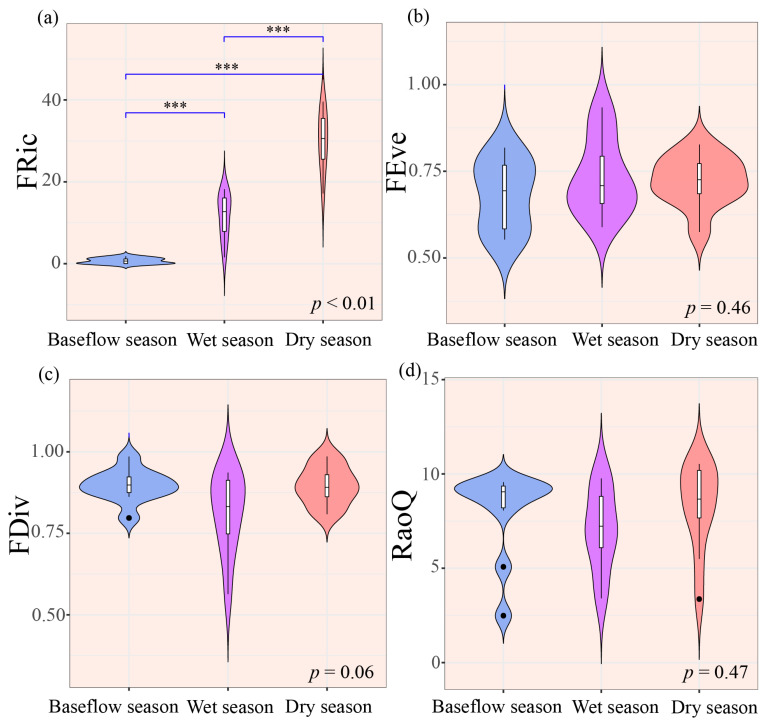
Seasonal variation in macroinvertebrate functional diversity indices. (**a**) FRic, (**b**) FEve, (**c**) FDiv, and (**d**) RaoQ. Note: In the box plot, the central horizontal line represents the median value. The whiskers extend to 1.5× the interquartile range (IQR), and the box boundaries indicate the 25th and 75th percentiles. Significant differences are denoted by asterisks (*** *p* < 0.001).

Regarding functional diversity, only FRic varied significantly among the hydrological seasons (*p* < 0.01, Figure 4a). Moreover, FDiv and RaoQ paralleled taxonomic diversity, both reaching minima during the wet season (Figure 4c,d). Notably, FEve was significantly lower during the baseflow season (Figure 4b).

**Figure 5:**
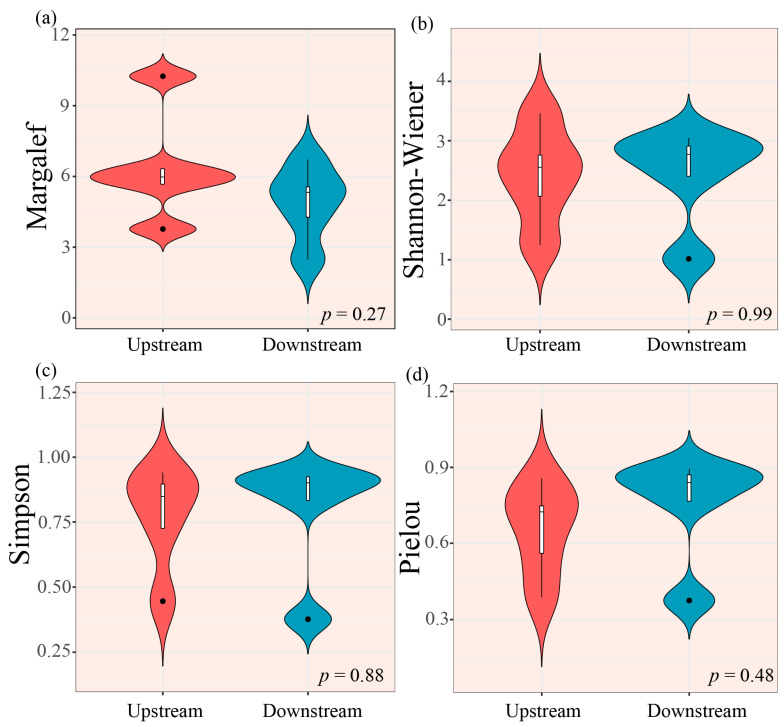
Longitudinal variation in macroinvertebrate taxonomic diversity indices. (**a**) Margalef richness index, (**b**) Shannon–Wiener diversity index, (**c**) Simpson diversity index, and (**d**) Pielou evenness index. Note: In the box plot, the central horizontal line represents the median value. The whiskers extend to 1.5× the interquartile range (IQR), and the box boundaries indicate the 25th and 75th percentiles.

Independent samples *t*-tests detected no significant upstream–downstream differences in any taxonomic diversity index (*p* > 0.05, Figure 5). However, the downstream areas showed elevated values for all indices except the Margalef richness index.

**Figure 6:**
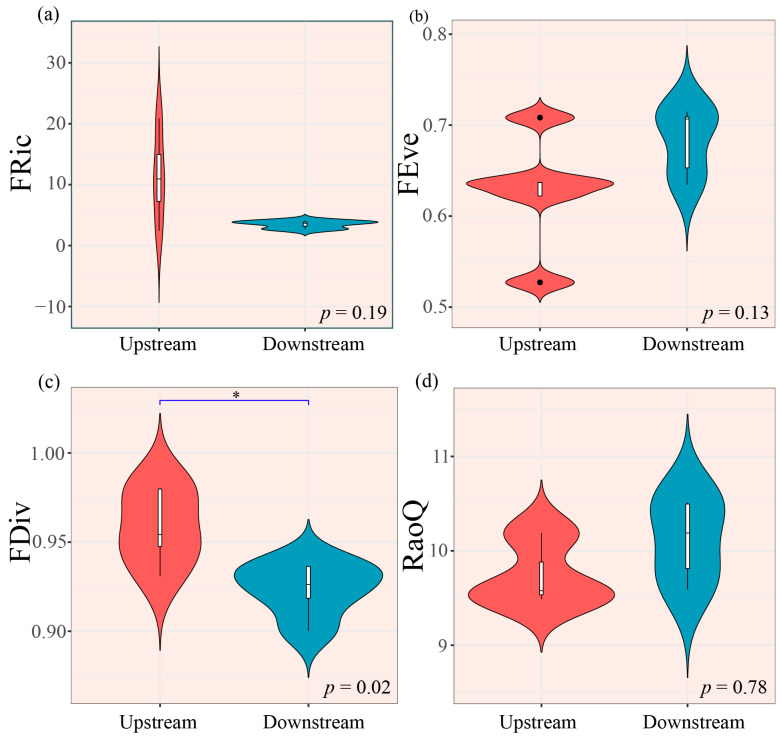
Longitudinal variation in macroinvertebrate functional diversity indices. (**a**) FRic, (**b**) FEve, (**c**) FDiv, and (**d**) RaoQ. Note: In the box plot, the central horizontal line represents the median value. The whiskers extend to 1.5× the interquartile range (IQR), and the box boundaries indicate the 25th and 75th percentiles. Significant differences are denoted by asterisks (* *p* < 0.05).

Functional diversity analyses only demonstrated significant spatial variation in FDiv (*p* < 0.01, Figure 6c). Specifically, the upstream areas exhibited a higher FRic (Figure 6a). Conversely, the downstream areas displayed elevated FEve (Figure 6b) and RaoQ (Figure 6d).

These findings suggest that taxonomic diversity was stable along the longitudinal gradient. In contrast, functional diversity responded strongly to reach-scale environmental variation.

### 3.3. Landscape Configuration Mediates Macroinvertebrate Biodiversity Patterns

We quantified the influences of landscape structure on macroinvertebrate diversity across the CSR using Mantel tests and random forest modeling (RF). Our results demonstrated significant spatial heterogeneity in the effects of landscape features on biodiversity. Specifically, landscape heterogeneity exerted a stronger influence on biodiversity in the upstream areas (12.2% of variance explained).

**Figure 7:**
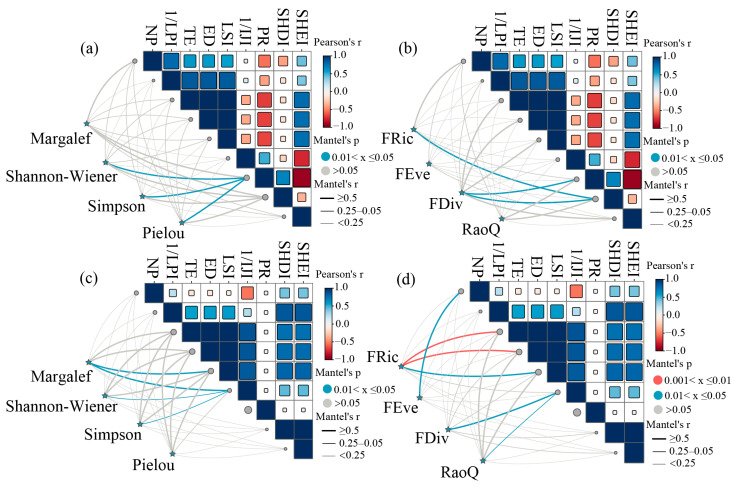
Relationships between macroinvertebrate diversity and landscape patterns in the CSR. (**a**) Taxonomic diversity indices in the upstream areas; (**b**) functional diversity indices in the upstream areas; (**c**) taxonomic diversity indices in the downstream areas; (**d**) functional diversity indices in the downstream areas. Note: FRic: functional richness index; FEve: functional evenness index; FDiv: functional divergence index; RaoQ: Rao’s quadratic entropy index; NP: number of patches; TE: total edge; ED: edge density; LSI: landscape shape index; LPI: largest patch index; IJI: interspersion juxtaposition index; PR: patch richness; SHDI: Shannon’s diversity index; SHEI: Simpson’s evenness index.

**Figure 8:**
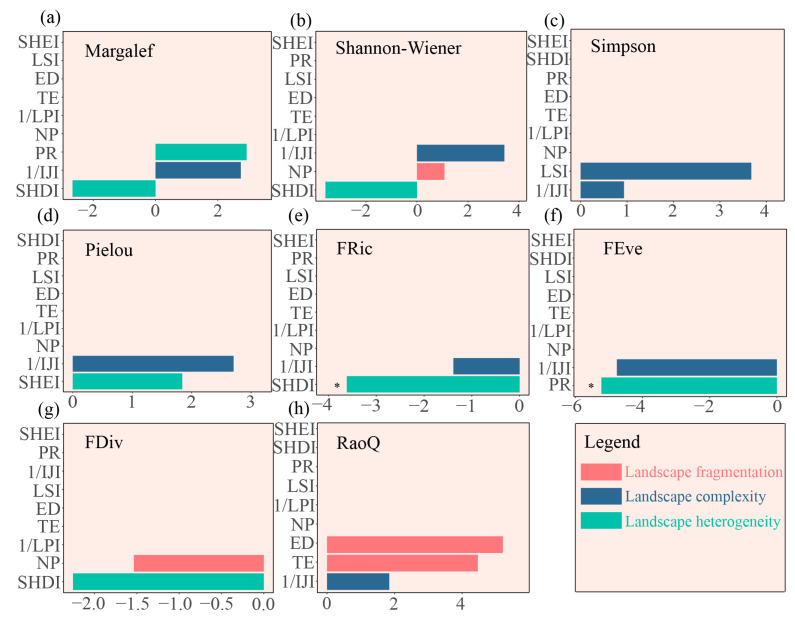
Importance of landscape pattern metrics for macroinvertebrate biodiversity in the upstream areas, as determined by RF analysis. The OOB error rate was 0.079, indicating high model accuracy. The OOB R^2^ was 0.81, indicating a strong predictive capability for biodiversity variation. (**a**) Margalef richness index, (**b**) Shannon–Wiener diversity index, (**c**) Simpson diversity index, (**d**) Pielou evenness index, (**e**) FRic, (**f**) FEve, (**g**) FDiv, and (**h**) RaoQ. Note: NP: number of patches; TE: total edge; ED: edge density; LSI: landscape shape index; LPI: largest patch index; IJI: interspersion juxtaposition index; PR: patch richness; SHDI: Shannon’s diversity index; SHEI: Simpson’s evenness index. Significant differences are denoted by asterisks (* *p* < 0.05).

Mantel tests indicated significant correlations between PR and macroinvertebrate taxonomic diversity in the upstream areas, including the Shannon–Wiener (r = 0.76, *p* = 0.03), Simpson (r = 0.75, *p* = 0.03), and Pielou indices (r = 0.70, *p* = 0.03) (Figure 7a). FRic was significantly associated with SHDI (r = 0.68, *p* = 0.04). Instead, FDiv exhibited strong positive associations with both SHDI (r = 0.90, *p* = 0.03) and PR (r = 0.88, *p* = 0.03) (Figure 7b). Furthermore, RF analysis corroborated these results, revealing that landscape heterogeneity indices (SHDI and PR) were the principal drivers of functional diversity in the upstream areas (Figure 8).

**Figure 9:**
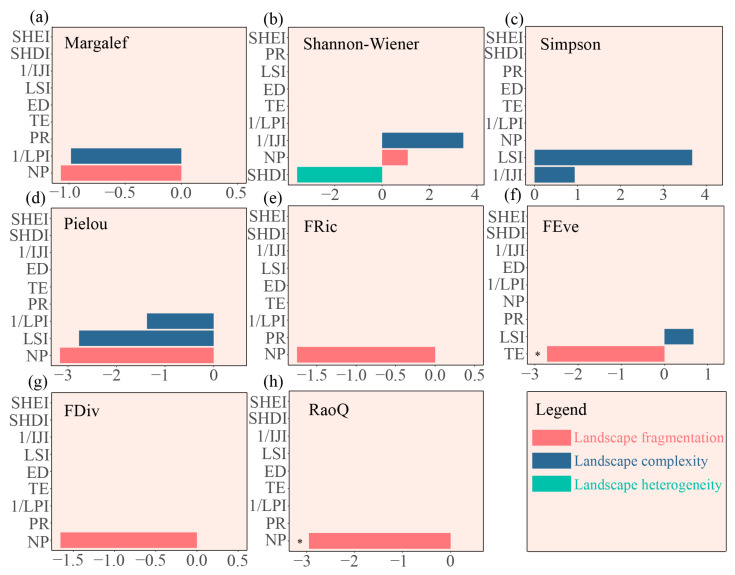
Importance of landscape pattern metrics for macroinvertebrate biodiversity in the downstream areas, as determined by RF analysis. The OOB error rate was 0.076, indicating high model accuracy. The OOB R^2^ was 0.79, indicating a strong predictive capability for biodiversity variation. (**a**) Margalef richness index, (**b**) Shannon–Wiener diversity index, (**c**) Simpson diversity index, (**d**) Pielou evenness index, (**e**) FRic, (**f**) FEve, (**g**) FDiv, and (**h**) RaoQ. Note: NP: number of patches; TE: total edge; ED: edge density; LSI: landscape shape index; LPI: largest patch index; IJI: interspersion juxtaposition index; PR: patch richness; SHDI: Shannon’s diversity index; SHEI: Simpson’s evenness index. Significant differences are denoted by asterisks (* *p* < 0.05).

By contrast, downstream area biodiversity was predominantly shaped by landscape fragmentation (10.3% of variance explained). Specifically, LSI correlated positively with Margalef richness (r = 0.73, *p* = 0.05) (Figure 7c). FRic showed strong positive associations with fragmentation metrics, including TE (r = 0.83, *p* < 0.01), ED (r = 0.83, *p* < 0.01), and LSI (r = 0.85, *p* = 0.02) (Figure 7d). In contrast, FEve was significantly correlated with NP (r = 0.51, *p* = 0.030). RF analysis further indicated that downstream functional diversity was predominantly influenced by landscape fragmentation metrics (TE and NP) (Figure 9).

Notably, the RF analysis revealed that taxonomic diversity exhibited no significant dependence on landscape drivers. This lack of association may reflect latitudinal variations in biodiversity responses to anthropogenic disturbance. Overall, our results reveal complex spatial relationships between macroinvertebrate biodiversity and landscape patterns across the CSR basin.

## 4. Discussion

### 4.1. Macroinvertebrate Community Dynamics Across Spatiotemporal Gradients

The CSR is the only remaining large tributary of the upper Yangtze River that retains a natural flow regime [26]. Systematic sampling identified 97 macroinvertebrate taxa, with Arthropoda (82.47%) dominating the community composition, followed by Mollusca (10.31%) and Annelida (7.22%). These findings align with previous studies that documented similar ecological patterns in the CSR [41]. Moreover, relative to major Yangtze tributaries (Yalong River [53] and Hanjiang River [54]), the CSR supports exceptionally high aquatic biodiversity.

Our results reveal pronounced spatiotemporal variation in the macroinvertebrate community composition. Taxonomic richness reached its minimum (54 species) during the wet season. This temporal pattern reflects the natural, dam-free hydrology of the CSR [39]. Seasonal floods impose pronounced hydrological disturbances, altering the substrate composition and habitat heterogeneity [27]. Therefore, these hydrodynamic changes likely explain the lower macroinvertebrate species richness observed during the wet season relative to the other hydrological seasons.

Spatially, maximum species richness (82 taxa) was recorded in the upstream areas, where assemblages were dominated by pollution-sensitive taxa (e.g., Ephemeroptera and Diptera). This pattern reflects the pristine conditions sustained by the complex alpine-valley topography of the Yunnan–Guizhou Plateau. This geomorphological complexity fosters diverse microhabitats while concurrently limiting anthropogenic disturbances [33]. In contrast, the downstream areas exhibited a significantly lower richness (61 taxa) and were dominated by pollution-tolerant taxa. This pattern correlates with the environmental modifications induced by extensive agricultural development in the riparian zone. Previous studies demonstrated that agricultural expansion within the downstream riparian zone has caused widespread vegetation loss and accelerated soil erosion [34]. Consequently, habitat degradation and environmental alteration have restructured macroinvertebrate assemblages. These findings partly account for the reduced macroinvertebrate richness observed in the downstream areas compared to the upstream areas. Our study emphasizes that maintaining natural hydrologic regimes and minimizing anthropogenic stressors are critical for preserving aquatic biodiversity.

The macroinvertebrate assemblages were dominated by Heptageniidae, Baetidae, Chironomidae, and Potamanthidae. Heptageniidae, Baetidae, and Chironomidae occurred consistently across all hydrological phases (baseflow, wet, and dry seasons) and along the entire upstream–downstream gradient.

The upper CSR is characterized by an alpine canyon topography with steep gradients and high-velocity flows under minimal anthropogenic disturbance. These conditions create an optimal habitat for rheophilic macroinvertebrates, including Heptageniidae and Baetidae. Chironomidae in the upstream areas were restricted to nearshore microhabitats with reduced flow velocities, which may be attributed to both physical habitat preferences and reduced interspecific competition in these areas [55]. Chi et al. [49] documented that riparian boulder formations created heterogeneous low-velocity microhabitats, supporting specific Chironomidae assemblages while also potentially providing increased food availability through organic matter retention. In contrast, the downstream areas exhibited elevated Chironomidae richness and higher abundances within depositional microhabitats characterized by reduced flow velocities. Here, both the sediment characteristics and enhanced nutrient supply may collectively contribute to these distribution patterns.

Mollusca constitute a key macroinvertebrate taxon in freshwater ecosystems [56]. Our study revealed that the high-elevation reaches were dominated by Basommatophora, including Physidae, Planorbidae, and Lymnaeidae. This assemblage composition aligns with earlier surveys [49]. These taxa exhibit specialized cold-adaptation mechanisms [55]. These adaptations enable population persistence in alpine environments.

### 4.2. Impacts of Seasonal and Spatial Variations on Macroinvertebrate Biodiversity

Mountain streams (e.g., the Chishui River) exhibit pronounced hydrological seasonality. As free-flowing systems without dam regulation, they experience substantial monsoonal flood disturbances during the rainy season [39]. These dynamic hydrological regimes generate heterogeneous habitat conditions that significantly influence macroinvertebrate assemblages and diversity [19].

The macroinvertebrate taxonomic diversity was significantly lower during the wet season relative to the other hydrological phases, as indicated by all four diversity indices (Figure 3). This observed pattern likely reflects substantial hydrological fluctuations associated with monsoon precipitation dynamics. Specifically, elevated precipitation regimes substantially alter stream discharge patterns. Such modifications subsequently affect macroinvertebrate assemblage structure and their spatial distribution. Concurrently, intensified hydrological variability induces scouring of fine sediments (e.g., silt and sand), while coarser substrates (e.g., gravel and cobblestones) remain more stable. This selective scouring contributes to habitat fragmentation, further constraining macroinvertebrate growth and reproduction [39]. These hydrological and habitat modifications collectively explain the observed reduction in macroinvertebrate taxonomic diversity during the wet season relative to other periods.

Trait-based functional diversity (FD) analyses provide critical insight into macroinvertebrate community ecology [57]. Previous studies have demonstrated that hydrological seasonality exerts a strong influence on functional diversity metrics [58,59]. Our results corroborate these earlier findings. Specifically, FRic exhibited pronounced seasonal variation, whereas taxonomic diversity remained temporally stable. Although community composition remained seasonally stable (PERMANOVA, *p* > 0.05), functional diversity indices such as FRic showed consistent seasonal patterns (ANOVA, *p* < 0.01; Figure 4a). This phenomenon suggests that macroinvertebrates respond to environmental variability primarily through functional traits (e.g., morphology, life history, and feeding habits) rather than taxonomic composition [60].

Organismal responses to hydrological seasonality are mediated by functional traits, including morphology, life history, and feeding habits [61]. These traits influence their stress-tolerance capacity, thereby determining taxon-specific distributions among hydrological phases. Environmental filtering under distinct flow regimes selectively alters trait assemblages, driving seasonal divergence in functional diversity metrics. Our findings strengthen the evidence that FD serves as a robust predictor of aquatic community responses to seasonal hydrological variability.

Previous studies have demonstrated that altitudinal gradients significantly influence macroinvertebrate FD [62,63]. High-altitude environments impose severe constraints on the distribution of macroinvertebrate species, pushing them to their physiological limits and exerting strong selective pressures on their traits [62]. For instance, species with “Univoltine” or “Semivoltine” life-history traits typically require stable habitats for growth and reproduction, whereas those with “Bi- or multivoltine” traits can persist in more variable environments [64]. This trait-based environmental filtering leads to systematic shifts in FD across elevations, explaining why FD spatial patterns in are more pronounced than those of taxonomic diversity.

The study area spans an elevational gradient from 300 m (downstream) to 1324 m (upstream). This gradient provides an ideal natural system to examine FD patterns along elevation gradients. Our results showed that FEve and RaoQ were significantly lower in the upper CSR. The reduced FEve indicates uneven resource utilization within macroinvertebrate communities [60]. This is consistent with previous reports of declining FEve under increasing anthropogenic disturbance [65].

Meanwhile, the reduced RaoQ values indicate decreased resource use efficiency among the macroinvertebrate communities [66]. This suggests the presence of intense interspecific competition. These findings indicate that communities in the upstream areas show inefficient resource utilization and intense competitive interactions. Such FD patterns may reduce community resilience to environmental disturbances. Consequently, regulating anthropogenic pressures in the upstream areas is essential for preserving macroinvertebrate biodiversity.

### 4.3. Landscape Patterns Influence Macroinvertebrate Biodiversity

Landscape patterns serve as critical indicators for assessing aquatic ecosystem health and biodiversity conservation [11]. Previous studies have demonstrated significant landscape-mediated effects on macroinvertebrate community biodiversity [24]. Our results highlight landscape heterogeneity as a primary determinant of FD patterns in macroinvertebrate communities in the upper CSR.

The upper CSR exhibits a complex topography characterized by mountains, hills, and deep valleys [33]. This landscape heterogeneity promotes FD by providing diverse habitats and ecological niches [21]. Landscape heterogeneity increases habitat type diversity. This, in turn, facilitates colonization by species with varied functional characteristics, thereby enhancing functional trait diversity within communities [67]. These processes collectively highlight the fundamental role of habitat heterogeneity in driving community assembly [68].

Biological communities in such heterogeneous landscapes are shaped by multiple environmental filters and species selection mechanisms [69]. These ecological mechanisms confer structural stability and increase community resilience to anthropogenic disturbances. Consequently, preserving landscape heterogeneity is a crucial strategy for conserving aquatic biodiversity.

The FD patterns of macroinvertebrates in the lower CSR are primarily driven by landscape fragmentation (10.3% of variance explained), contrasting with the upstream areas. Among various landscape metrics, fragmentation is a key determinant of aquatic biodiversity [24]. This influence operates through an increased number of discrete landscape patches, intensified edge effects, and reduced habitat connectivity [70].

Agricultural intensification in the lower CSR represents a principal driver of landscape fragmentation. Intensive farming practices have degraded riparian vegetation, thereby exacerbating soil erosion [34]. Meanwhile, urban and agricultural development have reduced habitat availability through fragmentation and loss [71]. This degradation of habitat quality threatens population viability, with documented declines in pollution-sensitive macroinvertebrates, particularly EPT taxa (e.g., Ephemeroptera and Trichoptera) in disturbed reaches [72]. Ecologically, fragmentation creates isolated patches that disrupt dispersal and gene flow. Simplified habitats reduce environmental heterogeneity, and both factors collectively weaken biodiversity maintenance mechanisms [68].

Collectively, these anthropogenic drivers highlight landscape fragmentation as a primary determinant of reduced FD in the heavily disturbed lower CSR. To maintain ecological integrity, we recommend region-specific regulatory measures, including (1) riparian buffer zone enforcement (e.g., ≥ 30 m vegetation buffers along waterways) and (2) pollution discharge limits aligned with macroinvertebrate conservation targets (e.g., EPT tolerance thresholds). These measures would mitigate habitat fragmentation in the downstream region of the CSR.

Interestingly, while the landscape configuration exerted a strong control on the FD metrics, we found no significant relationship with TD. These findings indicate that FD metrics are more sensitive than taxonomic approaches in detecting landscape-mediated ecological impacts. This enhanced responsiveness likely arises from the direct representation of organismal adaptation to environmental change by functional traits. Consequently, these findings underscore the necessity of integrating FD measures into landscape ecological assessments to fully capture biodiversity–ecosystem relationships.

While our study focused on landscape–biodiversity relationships, we recognize that unmeasured environmental variables (e.g., flow, water chemistry, and sedimentation) could potentially confound these relationships. Future studies incorporating these hydrologic and physicochemical measurements would help disentangle these complex interactions. Moreover, our sampling was conducted during a single year (2024), which may not capture the full range of interannual variability in macroinvertebrate communities and their relationships with landscape patterns. Future studies should prioritize multi-year monitoring programs to establish a more comprehensive understanding of the temporal dynamics in riverine biodiversity–landscape relationships.

## 5. Conclusions

This study revealed distinct spatial patterns in macroinvertebrate community composition and diversity across the CSR. Moreover, in the upstream areas, the functional diversity was primarily shaped by landscape heterogeneity. The complex topography created diverse ecological niches that filtered functional traits. Conversely, the downstream communities were predominantly influenced by landscape fragmentation. This influence occurred through mechanisms such as reduced habitat connectivity and intensified edge effects. Consequently, our findings highlight the critical importance of mitigating anthropogenic disturbances in the downstream areas and maintaining landscape diversity to preserve macroinvertebrate communities. This work advances our understanding of landscape–community interactions and provides a scientific foundation for biodiversity conservation. Future studies should incorporate midstream reaches to assess nonlinear responses along the river gradient.

## Data Availability

Data is contained within the article or Supplementary Materials.

## Supplementary Materials

The following supporting information can be downloaded at: https://www.mdpi.com/article/10.3390/biology14091149/s1, Table S1: Classification of macroinvertebrate traits; Table S2: Landscape metrics selected for this study and their ecological interpretations; Table S3: Dominant macroinvertebrate taxa across hydrological phases in the Chishui River; Table S4: Dominant macroinvertebrate taxa across longitudinal sections of the Chishui River; Table S5: PERMANOVA results for seasonal variation in macroinvertebrate community structure; Table S6: PERMANOVA results for spatial variation in macroinvertebrate community structure; File S1 (species list): List of macroinvertebrate species identified in the Chishui River.
