# Supplementary material for: Landscape Patterns Drive Functional Diversity of Macroinvertebrate Communities Along the Elevation Gradient in the Chishui River
Source: Biology (Basel). 2025 Aug 31;14(9):1149. doi: 10.3390/biology14091149 (PMC12467976; doi:10.3390/biology14091149)
Supplement: Supplementary file 1 [file biology-14-01149-s001.zip › Supporting Information.pdf]

Landscape Patterns Drive Functional Diversity of Macroinvertebrate Communities Along the Elevation Gradient in the Chishui River

Xiaopeng Tang [1], Zhenhao Liu [1], Fei Liu [2], Yun Cheng [3], Tingsong Yu [4], Xuehua Li [4], Qiang Qin [1] and Fubin Zhang [1,*]

[1] College of Environmental Science and Engineering, China West Normal University, Nanchong 637009, China; tangxiaopeng616219@163.com (X.T.); lzh18783900795@163.com (Z.L.); qiangqin710@163.com (Q.Q.)
[2] Institute of Hydrobiology, Chinese Academy of Sciences, Wuhan 430072, China; liufei@ihb.ac.cn
[3] Zhaoyang District Water Bureau, Zhaotong 657000, China; ztazj@126.com
[4] Yunnan Management and Conservation Bureau of National Nature Reserve of Rare and Endemic Fishes in the Upper Yangtze River, Zhaotong 657000, China; songeryu188@163.com (T.Y.); ynghjlxh@163.com (X.L.)
* Correspondence: sczhangfubin@163.com

Table S1

Classification of macroinvertebrate traits.

Trait	Trait state	Code
**Life history**		
Voltinism	Semivoltine (< 1 generation/year)	Volt1
	Univoltine (1 generation/year)	Volt2
	Bi- or multivoltine (> 1 generation/year)	Volt3
**Mobility**		
Occurrence in drift	Rare (catastrophic only)	Drif1
	Common (typically observed)	Drif2
	Abundant (dominant in drift samples)	Drif3
Swimming ability	None	Swim1
	Weak	Swim2
	Strong	Swim3
**Morphology**		
Attachment	None (free-ranging)	Atch1
	Some (sessile, sedentary)	Atch2
	Both	Atch3
Armoring	None (soft-bodied forms)	Armr1
	Poor (heavily sclerotized)	Armr2
	Good (e.g., some cased caddisflies)	Armr3
Shape	Streamlined (flat, fusiform)	Shpe1
	Not streamlined (cylindrical, round, or bluff)	Shpe2
**Ecology**		
Rheophily	Depositional only	Rheo1
	Depositional and erosional	Rheo2
	Erosional	Rheo3
Habit	Burrow	Habi1
	Climb	Habi2
	Sprawl	Habi3
	Cling	Habi4
	Swim	Habi5
	Skate	Habi6
Trophic groups	Collector-gatherer	Trop1
	Collector-filterer	Trop2
	Herbivore (scraper, piercer, and shedder)	Trop3
	Predator (piercer and engulfer)	Trop4
	Shredder (detritivore)	Trop5

Table S2

Landscape metrics selected for this study and their ecological interpretations.

Landscape pattern indices		Description
Landscape fragmentation	NP	The total number of patches within a certain landscape.
	TE	The total length of all patches of edges within a certain landscape (m).
	ED	The density of all patch edges within a certain landscape (m/hm^2).
Landscape complexity	1/LPI	LPI: Largest patch index. When the entire landscape consists of a single patch, 1/LPI converges to zero; conversely, as the area of the largest patch declines, 1/LPI asymptotically approaches unity.
	LSI	Landscape shape index used to measure the complexity of the category shape of the entire landscape.
	1/IJI	IJI: Interspersion and Juxtaposition index. Where the adjacency distribution among distinct patch types is maximally uneven, 1/IJI tends to unity; conversely, under perfectly even adjacency, 1/IJI approaches zero.
Landscape heterogeneity	PR	Patch richness, i.e., number of different patch types in the landscape.
	SHDI	Shannon's diversity index. Under a landscape comprising a single land-cover type, the index equals zero; with increasing number or proportional evenness of types, the index rises monotonically.
	SHEI	Shannon's evenness index. The metric approaches zero under maximally uneven patch distributions and converges to unity as patch distribution becomes increasingly uniform.

Table S3

Dominant macroinvertebrate taxa across hydrological phases in the Chishui River.

Group	Dominant taxa	Dominant degree		
		Normal season	Wet season	Dry season
Arthropoda	*Heptagenia* sp.	0.03	0.03	0.04
	Ameletus sp.	0.03		
	Ephemera sp.	0.03		
	Potamanthus sp.	0.10	0.11	0.22
	Baetis sp.	0.04	0.03	0.04
	Caenis sp.	0.03		
	Chironomidae larva	0.05	0.05	0.03
	Saldula sp.	0.03		
	Neocaridina denticulata		0.08	0.05

Table S4

Dominant macroinvertebrate taxa across longitudinal sections of the Chishui River.

Group	Dominant taxa	Dominant degree	
		Upstream	Downstream
Arthropoda	*Heptagenia* sp.	0.05	0.08
	Ameletus sp.	0.07	
	Ephemera sp.	0.05	
	Potamanthus sp.	0.38	
	Baetis sp.	0.04	0.07
	Caenis sp.		0.07
	Chironomidae larva	0.04	0.09
	Saldula sp.		0.17
	Neocaridina denticulata	0.05	0.09
Annelida	*Glossophonia* sp.	0.03	
Mollusca	*Corbicula fluminea*		0.03

Table S5

PERMANOVA results for seasonal variation in macroinvertebrate community structure.

	Df	Sum of squares	R^2	F	*p*-value
Group	2	0.83	0.08	1.16	0.24
Residual	27	9.67	0.92		
Total	29	10.50	1		

Table S6

PERMANOVA results for spatial variation in macroinvertebrate community structure.

	Df	Sum of squares	R^2	F	*p*-value
Group	1	0.76	0.29	3.32	0.01
Residual	8	1.83	0.71		
Total	9	2.59	1		
